# Epidemiology and Three-Month Recurrence of Tinea Infections in a Prison: A Cross-Sectional Study

**DOI:** 10.7759/cureus.101580

**Published:** 2026-01-15

**Authors:** Abdullah Abdullah

**Affiliations:** 1 General Medicine, Barts Health NHS Foundation Trust, London, GBR

**Keywords:** dermatophyte infection, environmental transmission, epidemiology, prison, recurrence, tinea

## Abstract

Background

Tinea infections are common dermatophyte infections with high recurrence rates, particularly in crowded institutional settings. Limited data exist on the epidemiology and recurrence patterns in prisons where environmental and behavioral risk factors are highly prevalent.

Objective

The objective of this study is to describe the epidemiology of tinea infections and characterize risk factor distribution in an incarcerated population with high recurrence rates.

Methods

A cross-sectional study was conducted among 73 inmates at a prison in Baghdad, Iraq. Patients with clinically diagnosed tinea infections were enrolled and followed for three months post-treatment. Baseline demographic, clinical, behavioral, and environmental data were collected. Univariate logistic regression and chi-square analyses were performed to evaluate potential predictors of recurrence.

Results

The overall three-month recurrence rate was 53.4% (39/73). Environmental risk exposures were nearly universal: 63 (86.3%) had a cellmate with tinea infection, 60 (82.2%) shared towels, and 56 (76.7%) shared razors. In univariate analysis, no individual-level factors significantly predicted recurrence, including age (OR 0.99, 95% CI 0.96-1.02, p=0.375), diabetes mellitus (OR 0.63, 95% CI 0.23-1.73, p=0.371), and prior tinea history (OR 0.61, 95% CI 0.24-1.54, p=0.296).

Conclusions

Tinea infections demonstrate extremely high recurrence rates (53.4%) in prisons despite standard treatment. The near-universal exposure to environmental risk factors and absence of individual-level predictors suggest that facility-level interventions, including provision of personal hygiene items, environmental disinfection, and concurrent treatment of contacts, may be more effective than targeting individual risk factors.

## Introduction

Dermatophyte infections, caused by keratinophilic fungi of the genera Trichophyton, Microsporum, and Epidermophyton, represent a significant global health concern. Current estimates indicate that approximately 20-25% of the world's population is afflicted by these infections, reflecting a considerable epidemiological burden that strains healthcare systems worldwide [1,2]. This high prevalence underscores the importance of enhancing awareness and management strategies globally, particularly in vulnerable populations such as those in correctional facilities.

The biology and pathogenesis of dermatophytes are primarily characterized by their unique enzymatic capabilities aimed at keratinized tissues, notably skin, hair, and nails. Dermatophytes employ various virulence factors, including keratinases and other proteases, which facilitate their invasion and colonization of host tissues by degrading the keratin found in these structures [3-5]. These virulence factors not only enable tissue degradation but also contribute to evasion of host immune responses, highlighting their role in the overall pathogenicity and chronicity of dermatophyte infections.

Prisons represent particularly high-risk environments for the spread of dermatophyte infections due to unique conditions inherent to closed settings. Overcrowding, limited ventilation, shared personal items, and barriers to healthcare access allow infections to spread rapidly among incarcerated individuals [6-10]. Such high rates often result from close quarters and compromised hygiene conditions that typically characterize correctional facilities, with profound implications for both inmate health and the broader public health landscape.

High recurrence rates of tinea infections pose substantial challenges to effective management strategies. Relapse is common after treatment, even with appropriate antifungal therapy [11]. The emergence of antifungal-resistant dermatophyte strains, particularly terbinafine-resistant Trichophyton species, poses additional challenges to effective treatment in institutional settings [2]. Furthermore, recurrent dermatophyte infections significantly impair quality of life, causing pruritus, social embarrassment, and psychological distress [12]. Untreated or recurrent infections in prisons can serve as reservoirs that may transmit to the larger community upon inmates' release [2].

While recurrence in tinea infections is well-documented in community settings, quantitative data on recurrence rates and risk factor patterns specifically in prison populations remain limited. Prison environments present unique epidemiological challenges, as environmental transmission dynamics differ substantially from community settings. In particular, it remains unclear whether traditional individual-level risk factors retain their predictive value in settings characterized by near-universal environmental exposure. Understanding these patterns has important implications for developing effective prevention and treatment strategies in correctional facilities.

The objective of this study was to describe the epidemiology of tinea infections and characterize factors associated with three-month recurrence in a prison population.

## Materials and methods

Study design and setting

This cross-sectional observational study was conducted at a prison in Baghdad, Iraq, between January 2023 and August 2023. The facility houses approximately 800 male inmates with varying sentence durations. The study protocol received institutional approval from the prison authority and the manager of the primary health care centre in the prison, and all participants provided verbal informed consent.

Participants and data collection

Male inmates aged 18 years or older with clinically diagnosed tinea infections were eligible for enrollment (N=73). Exclusion criteria included immunocompromised status (known HIV infection, organ transplant recipients, or immunosuppressive therapy), inability to complete three-month follow-up, concurrent systemic infections requiring hospitalization, and refusal to participate.

Baseline data collection included: (1) Demographics: age and diabetes mellitus status; (2) Clinical factors: prior tinea history, prior topical and systemic antifungal treatments, and baseline disease severity score; (3) Behavioral and environmental factors: shower frequency per week, sharing of towels and razors with other inmates, cellmate tinea infection status, and duration of imprisonment.

Patient selection

Consecutive sampling was employed during the study period. Inmates presenting to the prison health clinic with suspected tinea infections underwent clinical examination by a single physician (author name). Inclusion criteria required: (1) clinical diagnosis consistent with dermatophyte infection based on characteristic morphology and distribution, (2) age ≥18 years, (3) ability to attend three-month follow-up visit, and (4) provision of informed consent. Exclusion criteria were known HIV infection, current immunosuppressive therapy, concurrent systemic infections requiring hospitalization, or planned transfer to another facility within the follow-up period.

Variable definitions

Disease severity was assessed using a three-level clinical severity score. A score of 1 (mild) indicated localized disease at one anatomical site, no nail involvement, and duration less than eight weeks. A score of 2 (moderate) indicated involvement of two anatomical sites, moderate symptoms, duration 8-24 weeks, and no nail involvement. A score of 3 (severe) indicated involvement of three or more anatomical sites, nail involvement (onychomycosis), duration exceeding 24 weeks, treatment resistance, or early relapse within three months. Shower frequency was recorded as the number of showers per week. Duration of imprisonment was measured in weeks from admission to the time of study enrollment.

Treatment protocol

All participants received standardized antifungal treatment according to anatomical site and severity. Topical therapy consisted of clotrimazole 1% cream applied twice daily for 2-4 weeks. Systemic therapy (for extensive or refractory cases) consisted of terbinafine 250 mg orally once daily for 2-4 weeks [13]. At the time of this study (January-August 2023), terbinafine remained the available systemic antifungal agent in Iraq based on local availability. While emerging resistance has been reported in India and other regions [2], no routine susceptibility testing was available at our facility. The choice of terbinafine reflects standard care practices in the study setting.

Follow-up and outcome assessment

Participants were clinically assessed at three months post-treatment completion. The primary outcome was recurrence of tinea infection at any anatomical site, defined as either recurrence at the original infection site after documented clinical cure, or new tinea infection at a different anatomical site.

Missing data

No participants were lost to follow-up. All 73 enrolled participants completed the three-month assessment. Data completeness was 100% for all analyzed variables, as baseline assessments were conducted by structured interview and direct examination at enrollment.

Statistical analysis

Data were analyzed using Jamovi (version 2.x). Descriptive statistics were calculated for all variables. Continuous variables were expressed as mean ± standard deviation and compared using independent t-tests. Categorical variables were presented as frequencies and percentages (n, %) and compared using chi-square tests; chi-square statistics (χ²), degrees of freedom (df), and Cramér's V effect sizes are reported. Univariate logistic regression analyses were performed to calculate odds ratios (ORs) with 95% confidence intervals (CIs) for potential predictors. p-values were derived from Wald chi-square tests. Statistical significance was defined as a two-tailed p-value less than 0.05.

Covariate selection

Covariates for univariate analysis were selected a priori based on biological plausibility and prior literature documenting associations with dermatophyte infections. These included host factors (age, diabetes mellitus, prior tinea history, baseline severity), behavioral factors (shower frequency), and environmental exposures (shared personal items, cellmate infection status, duration of imprisonment).

## Results

Participant characteristics

Seventy-three male inmates with clinically diagnosed tinea infections completed the three-month follow-up and were included in the final analysis. The mean age was 40.7 ± 14.1 years (range: 19-64 years). Diabetes mellitus was present in 22 (30.1%) participants. Prior tinea history was reported by 36 (49.3%) participants.

Environmental risk exposures were nearly universal in this population: 63 (86.3%) had a cellmate with known tinea infection, 60 (82.2%) shared towels, and 56 (76.7%) shared razors. Mean duration of imprisonment was 23.2 ± 15.7 weeks. Baseline characteristics stratified by relapse status are presented in Table 1.

**Table 1:** Baseline Characteristics by Three-Month Relapse Status SD: standard deviation; χ²: chi-square statistic; df: degrees of freedom; V: Cramér's V effect size. *t-test used for continuous variables.

Characteristic	No Relapse (n=34)	Relapse (n=39)	χ²	df	V	p-value
Age (years), mean ± SD	42.3 ± 13.6	39.4 ± 14.5	-	-	-	0.375*
Diabetes mellitus, n (%)	12 (35.3)	10 (25.6)	0.804	1	0.105	0.37
Prior tinea history, n (%)	19 (55.9)	17 (43.6)	1.1	1	0.123	0.295
Shower freq/week, mean ± SD	2.79 ± 0.81	2.92 ± 0.84	-	-	-	0.501*
Shared towels, n (%)	30 (88.2)	30 (76.9)	1.59	1	0.148	0.208
Shared razors, n (%)	27 (79.4)	29 (74.4)	0.26	1	0.06	0.61
Cellmate with tinea, n (%)	31 (91.2)	32 (82.1)	1.28	1	0.132	0.258
Severity score, mean ± SD	2.71 ± 0.58	2.62 ± 0.59	-	-	-	0.507*
Duration (weeks), mean ± SD	24.1 ± 15.7	22.5 ± 15.9	-	-	-	0.672*

Three-month recurrence rate and univariate analysis

The overall three-month recurrence rate was 53.4% (39/73; 95% CI: 41.4%-65.0%). Univariate logistic regression analysis revealed no statistically significant predictors of recurrence. Complete univariate results are presented in Table 2.

**Table 2:** Univariate Logistic Regression Analysis OR: odds ratio; CI: confidence interval.

Variable	OR	95% CI	p-value
Age (per year)	0.99	0.96 – 1.02	0.375
Diabetes mellitus	0.63	0.23 – 1.73	0.371
Prior tinea history	0.61	0.24 – 1.54	0.296
Cellmate with tinea	0.44	0.11 – 1.87	0.267
Shared towels	0.44	0.12 – 1.60	0.215
Shared razors	0.75	0.25 – 2.26	0.611
Shower frequency (per day/week)	1.22	0.69 – 2.14	0.501
Severity score	0.76	0.34 – 1.71	0.507
Duration of imprisonment (weeks)	0.99	0.97 – 1.02	0.672

## Discussion

This cross-sectional study documented an exceptionally high three-month recurrence rate (53.4%) for tinea infections in a prison population. Notably, no individual-level factors significantly predicted recurrence, suggesting that facility-level environmental factors may be the primary drivers of transmission and reinfection in this setting.

Universal environmental exposures

A key finding of this study is the near-universal prevalence of environmental risk exposures: 63 (86.3%) of participants had a cellmate with known tinea infection, 60 (82.2%) shared towels, and 56 (76.7%) shared razors. When exposure is nearly ubiquitous, individual-level risk factors become less relevant for predicting recurrence because virtually all individuals face similar environmental transmission pressure. This phenomenon, sometimes termed "saturation of exposure," may explain why none of the examined factors significantly predicted recurrence.

Absence of traditional risk factors

Contrary to expectations based on general dermatological literature, diabetes mellitus was not associated with increased recurrence risk (OR 0.63, p=0.371). In fact, diabetic patients showed a numerically lower recurrence rate: 10 (45.5%) of 22 diabetic patients relapsed compared to 29 (56.9%) of 51 non-diabetic patients. This unexpected finding may reflect unmeasured confounders, such as differential treatment intensity or closer medical monitoring of diabetic patients. Similarly, age showed no significant association with recurrence (OR 0.99, p=0.375), suggesting that age-related immune changes may be less relevant in the face of overwhelming environmental transmission.

Antifungal resistance considerations

The emergence of antifungal-resistant dermatophyte strains, particularly terbinafine-resistant Trichophyton species, poses additional challenges to effective treatment in institutional settings [2]. Although antifungal susceptibility testing was not performed in this study, the high recurrence rate observed may partly reflect resistance patterns, warranting further investigation in future studies.

Clinical and public health implications

The high recurrence rate and absence of individual-level predictors have important implications for prevention strategies in prisons. Traditional approaches focusing on identifying and treating "high-risk" individuals may be insufficient when environmental transmission is the primary driver. These findings suggest that facility-level interventions may warrant consideration, though prospective trials are needed to confirm efficacy. These interventions can include: provision of personal hygiene items to reduce sharing, environmental disinfection of shared facilities, concurrent treatment of cellmates and close contacts, mass screening and treatment programs, and improved access to showers and hygiene facilities [14].

The public health implications extend beyond the prison walls. Untreated or recurrent infections in prisons can serve as reservoirs that may transmit to the larger community upon inmates' release [2]. This potential for community transmission emphasizes the need for systematic screening and treatment programs specific to correctional facilities.

Strengths and limitations

Strengths of this study include a prospective three-month follow-up with standardized outcome assessment, comprehensive assessment of demographic, clinical, behavioral, and environmental factors, and a homogeneous population reducing socioeconomic confounding.

Limitations include the single-center design limiting generalizability to other prison systems or geographic regions, moderate sample size (n=73), potentially underpowering detection of smaller effects, clinical diagnosis without universal mycological confirmation, male-only population limiting generalizability to female inmates, and absence of treatment adherence data. Recall bias may have been introduced for self-reported behavioral variables such as prior tinea history and sharing practices. Selection bias may have occurred if inmates with more severe or persistent infections were more likely to present to the clinic. Residual confounding is likely present, as we did not adjust for potentially important factors such as nutritional status, concurrent medications, or co-morbid skin conditions. The observational nature of the study limits causal inference. The three-month follow-up period, while appropriate for short-term recurrence, may not capture delayed relapses or longer-term recurrence patterns. Additionally, the inability to distinguish true relapse from reinfection limits the interpretation of recurrence mechanisms. Finally, the lack of antifungal susceptibility testing prevents assessment of whether treatment failures reflected drug resistance versus environmental reinfection.

Treatment challenges in resource-limited settings

This study was conducted using standard care available in the prison health system, which limited our ability to modify treatment based on resistance patterns. The high recurrence rate (53.4%) may reflect multiple factors, including antifungal resistance, inadequate treatment duration, environmental reinfection, or suboptimal treatment adherence. The near-universal environmental exposure likely contributed substantially to recurrence regardless of treatment efficacy. Future studies should incorporate antifungal susceptibility testing and evaluate alternative treatment regimens, particularly in settings with documented terbinafine resistance.

## Conclusions

Tinea infections demonstrate extremely high recurrence rates (53.4%) in prisons despite standard antifungal treatment. The near-universal environmental exposures and absence of significant individual-level predictors preliminarily suggest that facility-level interventions warrant further investigation as potentially more effective approaches in reducing the burden of recurrent tinea infections in this vulnerable population.

Future research priorities include multi-center studies to confirm these findings, intervention trials testing facility-level prevention strategies, cost-effectiveness analyses of mass treatment approaches, molecular epidemiological studies to distinguish relapse from reinfection, and antifungal susceptibility testing to assess resistance patterns in this population.
